# In Vitro Comparison of Passive and Active Clinical Proton Beams

**DOI:** 10.3390/ijms21165650

**Published:** 2020-08-06

**Authors:** Anna Michaelidesová, Jana Vachelová, Jana Klementová, Tomáš Urban, Kateřina Pachnerová Brabcová, Stanislav Kaczor, Martin Falk, Iva Falková, Daniel Depeš, Vladimír Vondráček, Marie Davídková

**Affiliations:** 1 Nuclear Physics Institute of the Czech Academy of Sciences, Řež 130, 25068 Řež, Czech Republic; vachelova@ujf.cas.cz (J.V.); jana.klementova@img.cas.cz (J.K.); brabcova@ujf.cas.cz (K.P.B.); 2 Proton Therapy Center Czech, Budínova 2437/1a, 180 00 Prague, Czech Republic; vladimir.vondracek@ptc.cz; 3 Faculty of Nuclear Sciences and Physical Engineering, Czech Technical University in Prague, Břehová 7, 11519 Prague, Czech Republic; tomas.urban@fjfi.cvut.cz (T.U.); stanislavkczr@gmail.com (S.K.); 4 Institute of Molecular Genetics of the Czech Academy of Sciences, Vídeňská 1083, 14220 Prague 4, Czech Republic; 5 Institute of Biophysics of the Czech Academy of Sciences, Královopolská 135, 61265 Brno, Czech Republic; falk@ibp.cz (M.F.); ivafalk@ibp.cz (I.F.); depesd26@gmail.com (D.D.)

**Keywords:** proton therapy, pencil beam scanning, double scattering, cell survival

## Abstract

Nowadays, the irradiation methodology in proton therapy is switching from the use of passively scattered beams to active pencil beams due to the possibility of more conformal dose distributions. The dose rates of active pencil beams are much higher than those of passive beams. The purpose of this study was to investigate whether there is any difference in the biological effectiveness of these passive and active irradiation modes. The beam qualities of double scattering and pencil beam scanning were measured dosimetrically and simulated using the Monte Carlo code. Using the medulloblastoma cell line DAOY, we performed an in vitro comparison of the two modes in two positions along the dose–deposition curve plateau and inside the Bragg peak. We followed the clonogenic cell survival, apoptosis, micronuclei, and γH2AX assays as biological endpoints. The Monte Carlo simulations did not reveal any difference between the beam qualities of the two modes. Furthermore, we did not observe any statistically significant difference between the two modes in the in vitro comparison of any of the examined biological endpoints. Our results do not show any biologically relevant differences related to the different dose rates of passive and active proton beams.

## 1. Introduction

The clinical advantages of a proton beam were firstly suggested by Wilson in 1946 in his paper on the radiological use of high-energy protons [1]. Since the implementation of protons in cancer treatment in 1954 [2], an increased interest in proton therapy has been observed worldwide. The most important advantage of protons compared to high-energy photons, depositing their energy exponentially after a maximum located couple of centimeters inside the patient’s body, is that protons deposit most of their energy in a precisely defined depth of the patient’s body given by their initial kinetic energy. Their depth–dose distribution is called the Bragg curve, with a maximum known as the Bragg peak.

The first step in proton therapy is the generation of protons (hydrogen ionization) and their acceleration inside a particle accelerator, typically a cyclotron or a synchrotron. For treatment purposes, the beam needs to be spread longitudinally by superimposing beams of different energies and weights. To obtain a lower energy than the fixed energy coming out of the cyclotron, an adjustable amount of material must be placed into the beam path. This can be carried out by a degrader inserted right after the beam extraction or by placing a stack with a variable number of plates (range shifter), a plate with ripples (ridge filter), or a rotating wheel with an azimuthally changing thickness (range modulation wheel) inside the nozzle in the irradiation room. In the case of synchrotron, the energy is adjusted in the accelerator and therefore there is no need for any additional devices. This longitudinally spread proton beam is known as the Spread-Out Bragg Peak (SOBP) [3].

Along with the longitudinal spread, the beam has to be spread also laterally, which can be achieved by passive or active modes. Examples of passive modes are Single or Double Scattering (SiS or DS), while an example of an active mode is Pencil Beam Scanning (PBS). For the passive modes, the beam passes through the scatters (one or two, SiS or DS, respectively) and is subsequently spread in the lateral direction. Active modes are based on the use of scanning magnets, which redirect the narrow proton beam to several positions according to the treatment plan. The dose is then delivered to each layer of the volume spot by spot [3].

Currently, there is an increased interest in the use of active beam modes for proton therapy due to the possibility of delivering more conformal dose distributions to patients. Another advantage of the active irradiation modes is the production of lower levels of secondary-induced radiation, mostly neutrons produced by the proton beam interacting with the components of the technological constructions or patient-specific devices, such as collimators or compensators, which are not used in the case of the active modes [4,5]. Thus, the growing interest in active beams is not surprising, and the issue of the comparison of passive and active modes is very topical. 

The majority of biological studies on proton beams were performed using passive modes. The proton beam profiles can be adjusted to be almost identical, regardless of the mode used. However, Monte Carlo simulations on patients have shown considerably increased dose-averaged linear energy transfer (LET_d_) values at the distal fall-off of the proton beams in the case of the active beams compared to the passive beams, which can affect the biological response of tissues situated in this position during proton therapy [6]. Moreover, the dose rates of the active proton beams are several orders of magnitude higher than a few Gy/min for the passively scattered beams. Generally, high and ultrahigh dose rates are associated with reduced biological damage, which can be explained by oxygen depletion and radical recombination [7,8,9]. 

On the other hand, in more recent studies dealing with various dose rates of photon radiation, a decreasing cell survival has been found in AG01522 cells, unlike in more radioresistant DU145 cells, which stayed unaffected by the increasing dose rate [10]. More efficient cell killing with higher dose per pulse using 10 MV X-rays has been reported in the case of glioblastoma cell lines (T98G, U87-MG) [11]. Furthermore, studies on FLASH radiotherapy using ultrahigh dose rates of above 40 Gy/s confirmed an increase in the differential response in normal and tumor tissues [12,13,14].

For protons, Iwata et al. have found almost identical values of relative biological effectiveness (RBE) in SOBPs formed by passive and active irradiation modes [15], and a comparison of a continuous beam with an ultra-high dose rated beam (10 MGy/s) produced by laser acceleration displayed only a statistically insignificant increase in the relative RBE in the latter case [16]. However, the irradiation of HLE cells by active proton beams caused a decrease in DNA synthesis, unlike the irradiation by passive beam [17]. It has also been observed that the delivery of higher doses per pulse can reduce cell survival due to an increase in the DNA damage, mostly in the yield of double-strand breaks (DSB) [18]. Other studies suggest that the higher dose rates can induce modifications of proteins, fatty acids, and cell organelles in the cytoplasm, which can lead to cell death [19,20,21]. 

The aim of the present study was to compare the influence on the biological response on different dose rates associated with the PBS (active) and DS (passive) modes at the Proton Therapy Center Czech (PTC). PTC is equipped with an isochronous cyclotron Proteus-235 (Ion Beam Applications, IBA, Belgium), which accelerates protons to an energy of 230 MeV. The comparison was held to investigate several biological endpoints after irradiation in two different positions in Bragg curves with similar dosimetric properties in both irradiation modes. The beam properties were simulated using the Monte Carlo code to reveal possible differences in the beam qualities. 

## 2. Results

### 2.1. Monte Carlo Simulations

**Figure 1:**
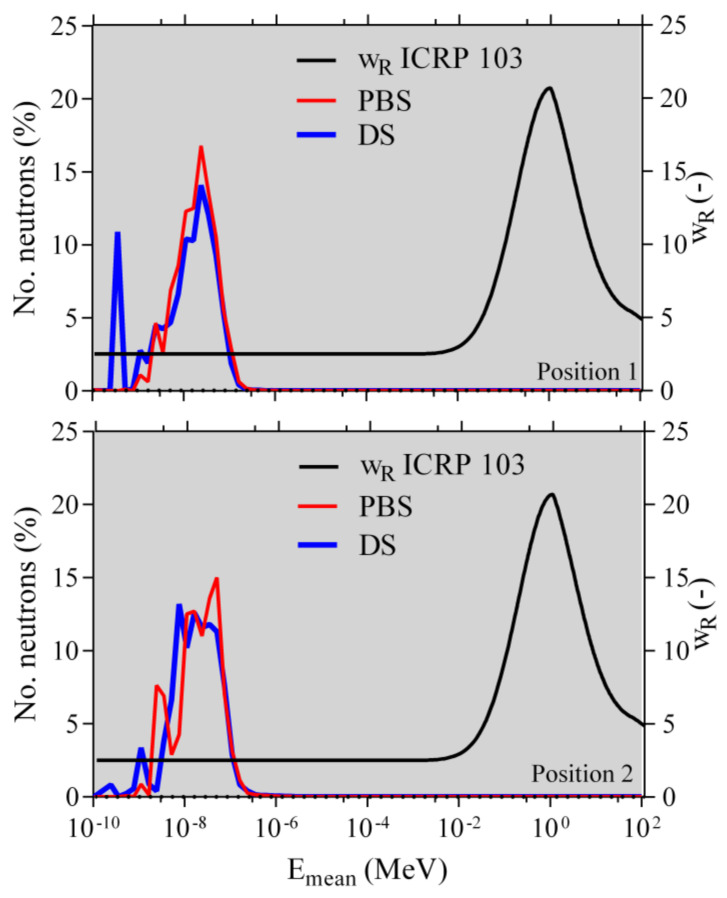
The normalized neutron spectra (the total number of neutrons is taken as 100%, as is shown in Table 2 and Table 3) and the representation of the radiation weighting factors (w_R_) according to the ICRP Publication 103 [22].

The LET_d_ values for protons, electrons, positrons, deuterons, tritons, helions, and alphas as well as the LET_d_ values of all these particles together for the two modes (PBS, DS) and the two positions (Position 1 and Position 2) are given in Table 1. Furthermore, in Table 2 and Table 3, information about the neutron production for the two modes and the two positions is provided. In Figure 1, the calculated neutron spectra and the radiation weighting factors (w_R_) according to the Publication 103 of the International Commission on Radiological Protection (ICRP) [22] are shown.

### 2.2. Cell Survival 

**Figure 2:**
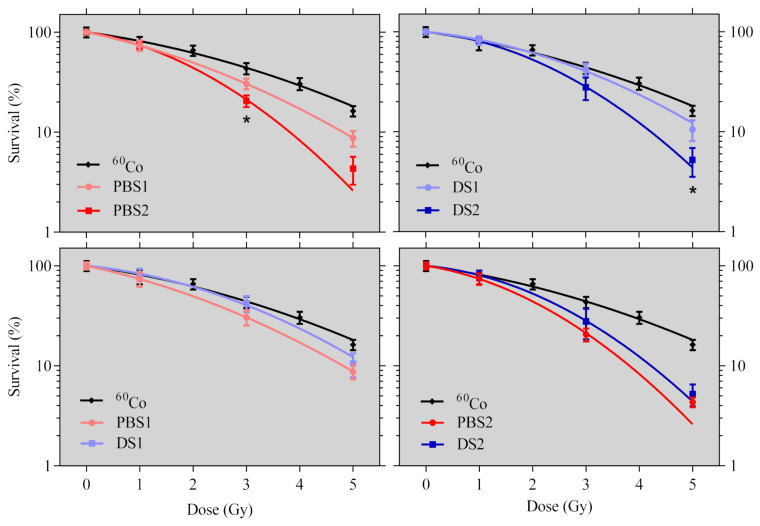
Clonogenic cell survival data of cells irradiated by Pencil Beam Scanning (PBS) and Double Scattering (DS) proton modes in the two irradiation positions (1—plateau, 2—Bragg peak) and by a ^60^Co gamma source. Up: a comparison of the two positions for each of the modes. Down: a comparison of the two modes for each of the positions. Points correspond to the experimental averages, with their standard deviations and lines corresponding to the LQ model fit. The asterisk (*) corresponds to *t*-tests lower than 0.05.

The parameters of the Linear-Quadratic (LQ) model and the cell survival data from the proton irradiations and those from the irradiation by a ^60^Co source are presented in Table 4 and Figure 2. 

### 2.3. Apoptosis

**Figure 3:**
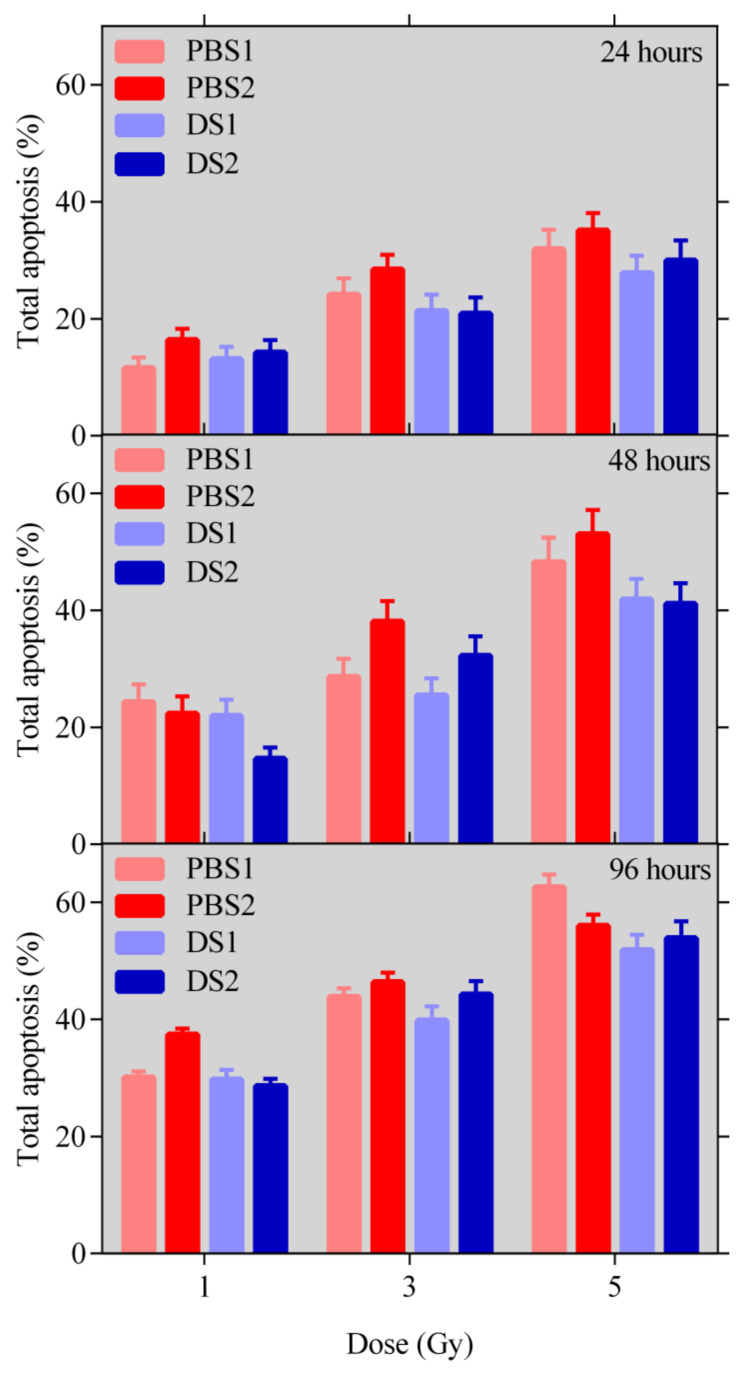
The percentage of Caspase 3/7-positive cells undergoing apoptosis (total apoptosis = early + late) determined for the two modes and two positions at three different periods of time post irradiation. No statistically significant differences have been found using the *t*-test.

The percentages of total apoptotic cells for the three different periods of time post irradiation after deducting the percentages of apoptosis in the control samples are shown in Figure 3. The total apoptosis includes cells (Caspase 3/7-positive) undergoing both early and late apoptosis. 

### 2.4. Micronuclei Assay

**Figure 4:**
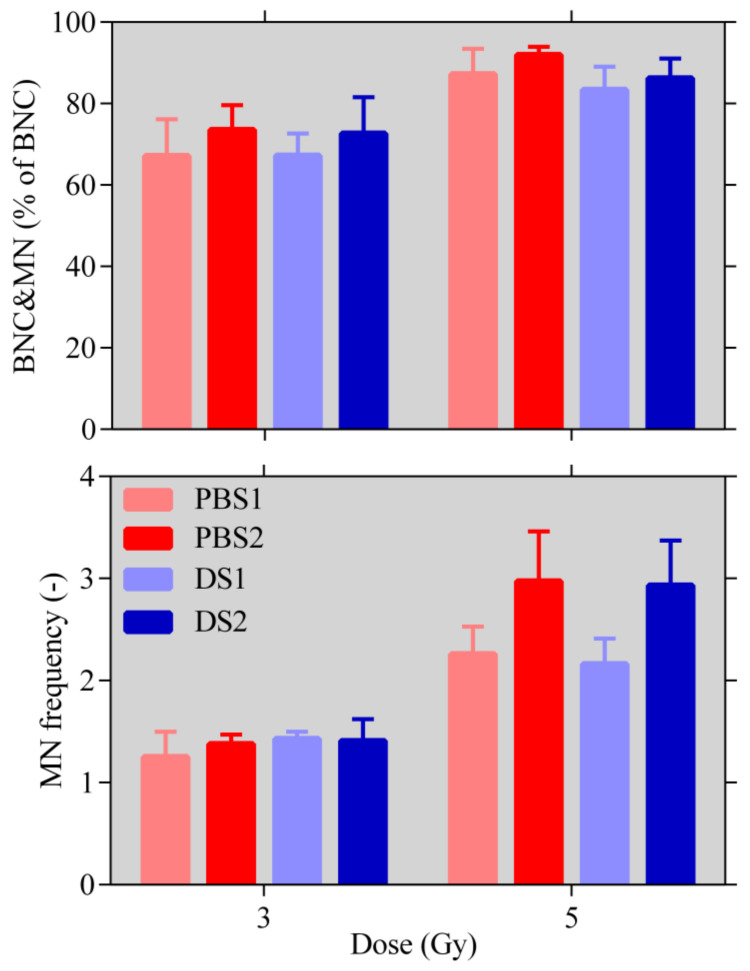
The percentage of binuclear cells (BNC) containing micronuclei (MN) from the total number of BNC and the micronuclei frequencies for the two modes and the two positions in the proton beam for doses of 3 and 5 Gy. No statistically significant differences have been found using the *t*-test.

The average percentages with their standard deviations of binuclear cells (BNC) containing micronuclei (MN) are shown on the upper side of Figure 4 for both modes and both positions in the Bragg peak. On the lower side of Figure 4, the micronuclei frequencies are given.

### 2.5. γ H2AX Assay

**Figure 5:**
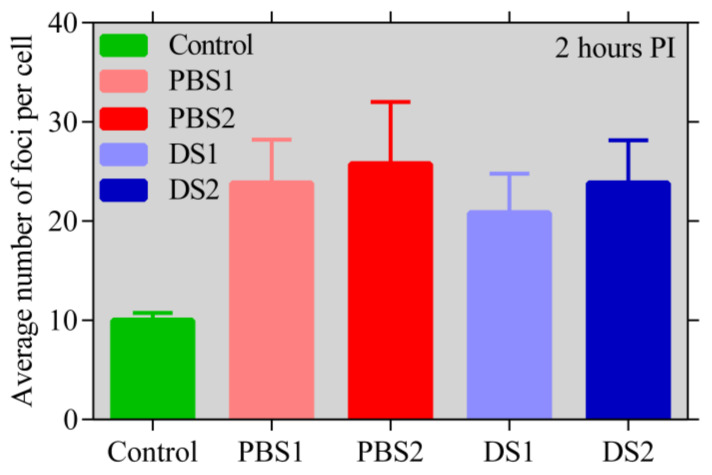
The average numbers of γH2AX foci per nucleus with their standard deviations compared for the two modes and the two positions in the proton beam path; measured two hours post irradiation by dose 1 Gy. No statistically significant differences have been found using the *t*-test.

The average number of γH2AX foci two hours after the irradiation is shown in Figure 5.

## 3. Discussion

The comparison of the two modes of proton beam irradiation has been performed using the medulloblastoma cell line DAOY and several selected biology endpoints. The DAOY cell line has been selected as a representative brain/cerebellum tumor highly relevant from the clinical point of view. The inclusion of another cell line in the study would be important to obtain more general results, but on the other hand would further decrease the statistical relevance of our results, since we have used the maximum beam time available. All the irradiations and sample processing have been repeated in two independent experiments. The independent runs have been realized with a time separation of several months to exclude unpredictable effects of the cell culturing or accelerator performance. Cryopreserved cells have been defrosted and cultured to comparable low passages prior to experimental processing in each run, and several sample replicates for each dose/time point and the irradiation method have been used. Although we are not able to extend further the present study for more independent experimental repeats or different cell lines, we believe our manuscript presents relevant knowledge when it is interpreted in combination with other studies.

In Table 1, the simulated LET_d_ values for protons, electrons, positrons, deuterons, tritons, helions, and alphas as well as the LET_d_ values for all the particles together for the two modes (PBS, DS) and two positions (Position 1 and Position 2) are presented. The LET_d_ values do not manifest any significant differences between the two modes. The same can be stated for neutrons. A summary of the neutron production along the primary beam path is given in Table 2 and Table 3. In the first position, a higher number of neutrons is produced. While looking at Figure 1, we can observe that almost all the produced neutrons are located in a region where the w_R_, according to the ICRP 103, is equal to 2.5. This means that the produced neutrons from both modes in both positions are biologically almost identical, with not a very high biological relevance. We thus believe that we can consider the beam qualities of both modes to be the same, and possible biological differences between the two modes must be assigned to the different dose rates. From the information provided by IBA staff about the current from the cyclotron, the beam line efficiency, and other beam parameters, we could calculate that the dose rate in the PBS mode is about 4000 times higher than that in the DS mode.

The data fitted by the LQ model have revealed statistically significant differences in cell survival between the two positions for each mode (Figure 2 upper side, Table 4). However, no statistically significant differences have been found between the two modes, despite the fact that a slightly lower survival for the PBS mode is detectable in both irradiation positions (Figure 2 lower side, Table 4). Using the α and β parameters estimated from the LQ model fit, the RBE values in comparison to ^60^Co have been estimated for both modes and both positions at the 50% cell survival level. The RBE_50%_ for PBS1 was found to be equal to 1.34 ± 0.16; for PBS2 it was 1.48 ± 0.20, for DS1 it was 1.05 ± 0.16, and for DS2 it was 1.26 ± 0.16.

The apoptosis level and the number of BNC containing MN have been found to be almost identical for the two modes. In terms of micronuclei frequencies and the 5-Gy-dose point, the frequencies are higher for the peak position in comparison to the proximal position for both modes, which is in agreement with the expected more complex DNA damage at the peak position (see [23]). The micronuclei frequencies appear to be almost the same for the 3-Gy-dose point and slightly higher for PBS for the 5-Gy-dose point, but no statistically significant differences have been found using the *t*-test in both cases. 

Our results suggest that the in vitro biological effectiveness of PBS in comparison to DS is almost identical for doses above 1 Gy, even if the two modes have very different dose rates. This statement is based on the Monte Carlo simulations of the beams used for the biological study and on the results from all the studied biological endpoints (cell survival, apoptosis, γH2AX, and micronuclei assay). The combination of the MN test and γH2AX assay allowed us to sensitively monitor the effects of both lower and higher doses in parallel. While γH2AX is currently the most sensitive method to detect DSBs, the micronucleus test is much less sensitive and is inaccurate at low doses. As different doses and experimental approaches revealed the same trends, our results suggest that the radiation dose in the range studied does not influence the results, so we can jointly interpret our results irrespective of the dose. 

Our results are in agreement with a previously published study examining the differences in the PBS and DS in SOBP [15], where not only the dose rate, but also other effects can play a role in the biological comparison of the two modes. Since we used almost monoenergetic beams, our results show that the dose rate does not play any significant role in the in vitro biological effectiveness of protons (including secondary particles) in a dose range relevant for the proton therapy of cancer. 

## 4. Materials and Methods

### 4.1. Cell Culture

The used human medulloblastoma cells DAOY (ATCC^®^ HTB-186™, Manassas, VA, USA) were originally derived from a tumor in the posterior fossa of a four-year old male. The adherent DAOY cell line exhibited a doubling time of 24 h and a plating efficiency of 30%. 

The cells were grown in Improved Minimum Essential Medium (GIBCO, Gaithersburg, MD, USA) supplied with 10% fetal bovine serum (BIOSERA, Nuaille, France), 100 U/mL of penicillin, and 0.1 mg/mL of streptomycin (Sigma-Aldrich, St. Louis, MO, USA) in T25 culture flasks (TPP Techno Plastic Products AG, Trasadingen, Switzerland) placed in an incubator with a 5% CO_2_ atmosphere and a temperature of 37 °C. Non-synchronized cell populations were used for the irradiations to mimic the situation in the patients’ tissues better.

### 4.2. Proton Beam Description and Monte Carlo Simulations

The proton irradiations were performed using the PBS and DS modes at the PTC using depth–dose curves that were as similar as possible and had narrow energy spectra. We chose to use narrow energy spectra to achieve well-defined beams and maximal dose rates in both modes (in case of PBS, the goal was to irradiate the whole sample at once without the need to switch energies and thus to combine the total dose from more energy layers. Switching between energies would reduce the dose rate).

At first, the range of the DS mode was set to 23.9 g/cm^2^ and the modulation to 1.5 g/cm^2^, where modulation corresponds to the distance between the proximal and distal 90% dose on the percentage depth dose curve (PDD). The chosen modulation is the smallest and the chosen range is the highest available in clinics at PTC (there is a limitation in the combinations of range and modulation. It is not possible to use any range and any modulation, but the combination has to be selected from predefined options). No collimators or compensators at the nozzle exit were used to mitigate the low beam contamination by secondary particles.

**Figure 6:**
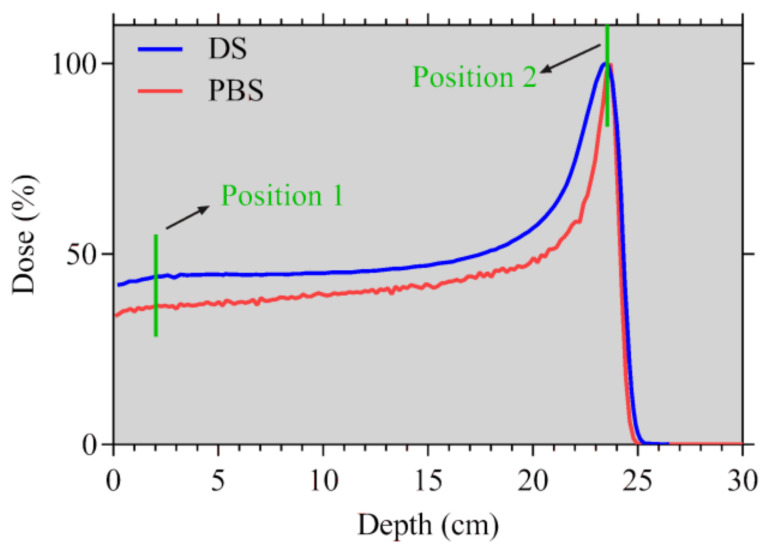
The percentage depth–dose curves for the selected beam energies for PBS and DS. The grey vertical lines symbolize the two irradiation positions of the cell cultures.

The selection of the high range allowed us to choose a high-energy beam for the PBS mode (190.6 MeV) and thus to obtain a wide Bragg peak, ensuring the reduced influence of potential sample positioning uncertainties. The PBS energy has been selected in order to ensure that both modes had the same range. The PDDs for both modes are shown in Figure 6.

Monte Carlo simulations have been performed to clarify especially secondary particle properties along the primary beam (in water phantom). The Monte Carlo code Fluka v. 2011.2x.6 [24,25] has been used for the estimation of the LET spectra of protons, light ions, electrons, and positrons, as well as the energy spectra of neutrons. All the simulations have been executed for the two modes in the simplified geometry of the treatment nozzles of these modalities.

The treatment nozzles, the water phantom, as well as the source term (e.g., proton beam) have been described by the appropriate tools of Fluka code. Firstly, the depth–dose distributions of the dose deposited in the water phantom for both modalities were simulated and compared with the experimental ones. The second step consisted of estimating the LET spectra of the charged particles and energy spectra of neutrons in sample irradiation positions along the beam path in the water phantom.

### 4.3. Analysis of the Monte Carlo Simulations

From the Monte Carlo simulations, we have obtained numbers of charged particles with a specific LET value. The dose-averaged LET (LET_d_) values were calculated from the raw data of the simulations using the following equation:

(1) $$LET_{d}=\frac{\sum_{i}\left({\hat{LET}}_{i}\cdot D_{i}\right)}{\sum_{i}D_{i}}$$

where $LET_{d}$ is the calculated dose-averaged LET in the specific position of the Bragg peak, ${\hat{LET}}_{i}$ is the LET value, and $D_{i}$ corresponds to the proportion of particles with this LET value in the specific position. 

In addition to the charged particles, the production of neutrons was also simulated. Since neutrons are uncharged particles, they do not have LET values. However, it was possible to generate the energy spectra of neutrons in each position of interest for each mode. Due to the fact that neutrons have, based on their energies, different biological consequences, they were further categorized based on the ICRP Publication 103 [22] to gain a better understanding of their biological consequences. 

### 4.4. Cell Sample Irradiations 

All the irradiations and sample processing were repeated twice (two independent experiments). The irradiations took place in two different positions along the Bragg curves, corresponding to 2 cm (Position 1) and 23.6 cm (Position 2) in water adjusted using water-equivalent RW3 and PMMA plates. These positions have been selected so that one of the series of samples has been in the plateau region and the second one in the peak. In the figures and tables in this paper, the two irradiation positions are marked as PBS1 and PBS2 or DS1 and DS2, respectively. The PBS and DS stand for the used mode, while 1 stands for the plateau (Position 1) and 2 for the peak position (Position 2).

The same doses were applied at each position and for each mode (DS, PBS), which means that the irradiations were executed in four parts (DS1, DS2, PBS1, PBS2). From Figure 6, it is evident that the PDD differs in the two modes and that the peak for DS mode is wider than that for PBS. The proximal position (Position 1, Figure 6) was implemented in the study to decrease the possibility of false positive results that could have been obtained from the measurements in the peak region, where small positioning uncertainties could have led to misleading results. Another advantage of analyzing two positions is the difference in the beam quality, which should be reflected in their different biological effects.

The doses for PBS have been established in the treatment planning system XiO by ELEKTA using plans with a spot spacing of 4 mm. The irradiation maps were constructed to enable the irradiation by 1, 3, and 5 Gy in the plateau and the peak region. The doses have been verified by a PPC05 ionization chamber (IBA Dosimetry GmbH, Schwarzenbruck, Germany) at the cell sample irradiation positions. 

As for the DS mode, the dose calibration and verification were performed using the same PPC05 ionization chamber to obtain the number of monitor units needed for each dose applied. The irradiation maps were created for both positions to get doses of 1, 3, and 5 Gy. The beam homogeneity was verified using the 2D LYNX detector (IBA Dosimetry GmbH, Schwarzenbruck, Germany) for all the created irradiation maps for both modes.

The irradiations were carried out in the gantry rooms of PTC, which provided the possibility of irradiating the samples through the irradiation table. This enabled us to place the samples horizontally, without the need to fill them completely with medium and thus to lower the stress of the cells. 

As for the cell survival, the micronuclei assay and the apoptosis assay the cells were irradiated in T25 flasks. In case of the γH2AX assay, the irradiation was performed in glass bottom dishes (SPL Life Sciences Co., Ltd., Pocheon-si, Korea). The cells were irradiated by doses of 1, 3, and 5 Gy for the cell survival and apoptosis assays. The samples for the γH2AX assay were irradiated by 1 Gy only to obtain a reasonable number of foci per cell for analysis (about 25–35 double-strand breaks, DSB, per cell nucleus [26]). In the case of the micronuclei assay, the cells were irradiated by doses of 3 and 5 Gy to obtain a number of micronuclei sufficiently higher than the micronuclei level of the control samples. 

The second set of samples was irradiated by doses of 1, 2, 3, 4, and 5 Gy using a ^60^Co γ-ray source at the Authorized Metrology Center of the Nuclear Physics Institute of the Czech Academy of Sciences (Prague, Czech Republic) to get reference cell survival curves. Due to the horizontal beam orientation, the cells were irradiated in a vertical position in T25 flasks fully filled with medium to avoid beam inhomogeneities and back scattering. The cell irradiations took place in a water depth of 5 cm ensured by a special holder inside a water tank placed at a source-surface distance of 75 cm. The irradiation field was restricted to an area of 10 × 10 cm^2^ by a primary and secondary collimator. The dose calibration measurements were performed in exactly the same geometry by a cylindrical PTW 30,013 ionization chamber (PTW, Freiburg, Germany). The dose rate was recalculated each time according to the irradiation date (around 0.01 Gy/s at the irradiation position), and the applied doses were achieved by increasing the time of irradiation according to the actual dose rate. The maximal positioning error was 2 mm, which corresponds to a dose uncertainty of less than 2% (calculated from the depth–dose curve obtained in the same geometry).

### 4.5. Post-Irradiation Sample Processing and Data Analysis

#### 4.5.1. Clonogenic Cell Survival Assay

Immediately after the irradiation, the cells were harvested (0.05% Trypsin by GIBCO or BIOSERA) and counted by the MUSE cell analyzer (EMD Millipore, Burlington, MA, USA) using the Muse Count and Viability Assay Kit (Merck Millipore-MCH100102), following the manufacturer’s instructions. The cells were then reseeded into 6-well plates for a clonogenic cell survival analysis. The seeded cell number was established according to the expected cell survival given by the delivered dose. The colonies formed nine days after irradiation were fixed and colored using crystal violet (Sigma-Aldrich, St. Louis, MO, USA) diluted in 95% methanol. 

The number of colonies in each well was counted manually, and the averages and standard deviations were calculated from every 6-well plate. Since there were two experimental runs, the averages and standard deviations were established from both runs for further processing. The survival data were fitted by the Linear-Quadratic (LQ) model [27] using the least square fitting procedure in the GraphPad Prism software (Version 7.0 for Windows, GraphPad Software, La Jolla, CA, USA, www.graphpad.com).

A statistical analysis of the data was performed as follows. To estimate if the variance of the two associated data sets was significantly different, the F-test from the one-way ANOVA was used. In cases where the F-value was lower than 0.05, the data sets were recognized to have different variances. According to the result of the F-test, the two tailed Student’s *t*-test with the same or different variance was used to estimate the statistical significance of the difference between the evaluated datasets. The data were considered to be significantly different if the *p*-value from the *t*-test was lower than 0.05, as visualized in the graphs using an asterisk (*). The same statistical analysis was performed for the other biological endpoints as well.

#### 4.5.2. Apoptosis Measurement

The cell medium was replaced immediately after the irradiation with a fresh one. The level of apoptosis was established in samples 24, 48, and 96 h post irradiation based on the activation of Caspase 3/7 using the MUSE^™^ Caspase-3/7 Kit (Millipore-MCH100108) according to the manufacturer’s instructions. Duplicates were prepared for each examined situation. Since there were two experimental runs, the averages and standard deviations were then established from the two runs.

#### 4.5.3. Micronuclei Assay

The irradiated cells were reseeded at a 30% confluence in dishes with glass coverslips. Twenty-four hous later, cytochalasin B (Sigma-Aldrich, St. Louis, MO, USA) in a concentration of 1 μg/mL of media was added. After another 24 h, the cells were fixed with 4% paraformaldehyde and stained by 4,6-diamidine-2-phenylindole dihydrochloride (DAPI) (Sigma-Aldrich, St. Louis, MO, USA). The images were captured at the CZ-OPENSCREEN center (Institute of Molecular Genetics of the CAS, Prague, Czech Republic) by the Operetta High-Content Imaging System (Version HH12940101, PerkinElmer, Waltham, MA, USA) at 40× magnification using the Harmony software (Version 4.1, PerkinElmer, Waltham, MA, USA).

A minimum of 50 fields per slide were analyzed using in-house software following the criteria of the HUMN project [28] written in PYTHON. The binuclear cells (BNC) containing micronuclei (MN) and the total number of MN were scored. The MN frequencies were calculated as the total number of MN divided by the total number of BNC in each sample. Duplicates were prepared for each dose point. The averages and standard deviations were established from the two experimental runs. 

#### 4.5.4. γ H2AX Assay

DSBs are the most serious lesions induced in the DNA molecule by ionizing radiation [29]. DSBs contribute mostly to cell death and/or mutagenesis. The detection of γH2AX foci and/or other DSB repair foci currently represents the most sensitive method for their scoring [30,31]. 

Briefly, the cells were grown and irradiated in dishes with glass coverslips and fixed two hours post irradiation by 4% paraformaldehyde for 10 min at room temperature. The cells were permeabilized using 0.1% Triton X-100. Consequently, they were treated by 5% bovine serum albumin (BSA) solution (Sigma-Aldrich, St. Louis, MO, USA) before being incubated with the primary antibodies. The primary mouse anti-H2AX antibody (Millipore) diluted in 1% BSA was added and the cells were incubated overnight at 4 °C. On the next day, the samples were washed three times in 0.1% BSA and the staining was visualized by goat anti-mouse IgG ALEXA Fluor 488-conjugated secondary antibody (Invitrogen, Carlsbad, CA, USA) diluted in 1% BSA. After a repeated washing with 0.1% BSA, the nuclei were stained with DAPI. 

For this “screening” analysis with large numbers of analyzed cells, the time point of two hours post irradiation was selected as a result of a compromise. The maximum number of foci is found in irradiated cells 15–30 min post irradiation [25,32]; however, after the two hours the number of foci is still high enough to allow statistically relevant comparisons with lower time expenses required for focus counting. 

The images were captured at the CZ-OPENSCREEN center (Institute of Molecular Genetics of the CAS) by the Operetta High-Content Imaging System (Version HH12940101, PerkinElmer, Waltham, MA, USA) at 40× magnification using the Harmony software (Version 4.1, PerkinElmer, Waltham, MA, USA). A minimum of 100 fields per slide were analyzed by the Columbus image analysis software (Version 2.7.1, PerkinElmer, Waltham, MA, USA), which enables the detection and counting of fluorescently stained γH2AX foci in the DAPI-stained nuclei of cells. Duplicates were prepared for each time point. The averages and standard deviations were established from the two experimental runs.

## 5. Conclusions

Our results demonstrate that the dose rate does not significantly influence the biological effectiveness of clinical proton beams (including the effect of secondary particles produced) for doses of above 1 Gy. We have found that the PBS and DS modes in proton therapy can be considered as mutually equivalent concerning the biological responses of irradiated cells.

## Figures and Tables

**Table 1:** The dose-averaged linear energy transfer (LETd) values for the two modes and the two positions.

	LET_d_ (keV/µm)			
	Position 1		Position 2	
	PBS	DS	PBS	DS
Protons	0.45	0.45	3.17	3.43
Electrons	0.28	0.28	0.19	0.19
Positrons	0.19	0.19	0.18	0.18
Deuterons	5.74	5.57	8.68	7.40
Tritons	7.54	8.27	8.08	11.16
Helions	32.00	32.80	59.73	42.79
Alphas	51.79	51.76	102.58	103.00
All particles	0.44	0.44	3.11	3.34

**Table 2:** Summarized neutron production at Position 1 and their categorization according to the ICRP 103 report [22].

E (MeV)	No. PBS (-)	No. DS (-)	No. PBS (%)	No. DS (%)
≤1	4.36 × 10^8^	6.20 × 10^8^	1.00 × 10^2^	1.00 × 10^2^
1–50	2.61 × 10^1^	3.74 × 10^1^	5.99 × 10^−6^	6.03 × 10^−6^
≥50	6.81 × 10^0^	7.70 × 10^0^	1.56 × 10^−6^	1.24 × 10^−6^
All energies	4.36 × 10^8^	6.20 × 10^8^	1.00 × 10^2^	1.00 × 10^2^

**Table 3:** Summarized neutron production at Position 2 and their categorization according to the ICRP 103 report [22].

E (MeV)	No. PBS (-)	No. DS (-)	No. PBS (%)	No. DS (%)
≤1	2.94 × 10^8^	2.63 × 10^8^	1.00 × 10^2^	1.00 × 10^2^
1–50	1.33 × 10^2^	1.21 × 10^2^	4.52 × 10^−5^	4.61 × 10^−5^
≥50	2.67 × 10^1^	2.44 × 10^1^	9.09 × 10^−6^	9.27 × 10^−6^
All energies	2.94 × 10^8^	2.63 × 10^8^	1.00 × 10^2^	1.00 × 10^2^

**Table 4:** The parameters of the Linear-Quadratic (LQ) model (α, β), their standard errors (σ_α_, σ_β_), and the correlation parameter R^2^.

Data Set	α (Gy^−1^)	σ_α_ (Gy^−1^)	β (Gy^−2^)	σ_β_ (Gy^−2^)	R^2^
^60^Co	0.17	3.25 × 10^−2^	0.03	9.99 × 10^−3^	0.99
PBS1	0.26	1.13 × 10^−3^	0.05	2.77 × 10^−2^	1.00
PBS2	0.20	3.95 × 10^−4^	0.11	1.27 × 10^−2^	1.00
DS1	0.13	2.87 × 10^−2^	0.06	9.49 × 10^−3^	1.00
DS2	0.12	1.21 × 10^−2^	0.10	4.99 × 10^−3^	1.00
